# Distinctive PSA-NCAM and NCAM Hallmarks in Glutamate-Induced Dendritic Atrophy and Synaptic Disassembly

**DOI:** 10.1371/journal.pone.0108921

**Published:** 2014-10-03

**Authors:** María Fernanda Podestá, Patricia Yam, Martín Gabriel Codagnone, Nonthué Alejandra Uccelli, David Colman, Analía Reinés

**Affiliations:** 1 Instituto de Investigaciones Farmacológicas (ININFA, CONICET-UBA), Ciudad Autónoma de Buenos Aires, Argentina; 2 Cátedra de Farmacología, Facultad de Farmacia y Bioquímica, Universidad de Buenos Aires, Ciudad Autónoma de Buenos Aires, Argentina; 3 Montreal Neurological Institute and Hospital, McGill University, Montreal, Canada; 4 Instituto de Biología Celular y Neurociencias “Prof. E. De Robertis” (IBCN, CONICET-UBA), Ciudad Autónoma de Buenos Aires, Argentina; INSERM U901, France

## Abstract

Dendritic and synapse remodeling are forms of structural plasticity that play a critical role in normal hippocampal function. Neural cell adhesion molecule (NCAM) and its polysialylated form (PSA-NCAM) participate in neurite outgrowth and synapse formation and plasticity. However, it remains unclear whether they contribute to dendritic retraction and synaptic disassembly. Cultured hippocampal neurons exposed to glutamate (5 µM) showed a reduced MAP-2 (+) area in the absence of neuronal death 24 h after the insult. Concomitantly, synapse loss, revealed by decreased synaptophysin and post-synaptic density-95 cluster number and area, together with changes in NCAM and PSA-NCAM levels were found. Dendritic atrophy and PSA-NCAM reduction proved NMDA-receptor dependent. Live-imaging experiments evidenced dendritic atrophy 4 h after the insult; this effect was preceded by smaller NCAM clusters (1 h) and decreased surface and total PSA-NCAM levels (3 h). Simultaneously, total NCAM cluster number and area remained unchanged. The subsequent synapse disassembly (6 h) was accompanied by reductions in total NCAM cluster number and area. A PSA mimetic peptide prevented both the dendritic atrophy and the subsequent synaptic changes (6 h) but had no effect on the earliest synaptic remodeling (3 h). Thus, NCAM-synaptic reorganization and PSA-NCAM level decrease precede glutamate-induced dendritic atrophy, whereas the NCAM level reduction is a delayed event related to synapse loss. Consequently, distinctive stages in PSA-NCAM/NCAM balance seem to accompany glutamate-induced dendritic atrophy and synapse loss.

## Introduction

Structural plasticity plays a crucial role in normal hippocampal function. Dendritic and synaptic remodeling in the *Cornus Ammonis* (CA) 1 and 3 regions, as well as neurogenesis in the *Dentate Gyrus* (DG) are the mechanisms involved in hippocampal structure reorganization [1], [2]. These neuroplastic phenomena enable hippocampal neurons to modify their synaptic morphology, which in turn allows them to adapt to new situations or environmental changes. It is worth noting that, if reorganization is not adequate, structural remodeling might contribute to nervous system malfunction [3]. Consequently, deregulation or disruption of this fundamental process may compromise the normal function of the hippocampus.

In line with this, dysfunctional hippocampal plasticity has been repeatedly evidenced in a number of clinical and preclinical studies. For instance, hippocampal volume reduction is presently one of the most consistent structural abnormalities found in schizophrenia, dementia, Alzheimer's, Parkinson's, depression and in other stress-related psychiatric disorders [4]–[6]. It has been clearly established that structural changes may affect synapse function. However, whether structural remodeling should be prevented or promoted in the aforementioned pathologies remains a matter of debate, since it may serve to prevent further damage. In this regard, alterations in somatodendritic, axonal and synaptic components have been proposed as putative mechanisms underlying hippocampal shrinkage [7]. Changes in spine morphology, dendritic atrophy and synapse loss have also been demonstrated in a number of the above mentioned disorders [8].

Numerous pieces of evidence strongly associate dendritic atrophy with the reduction in hippocampal volume, and with the hippocampal-dependent behavioral deficit characteristic of psychiatric and neurological disorders [9]–[12]. Glucocorticoids and excitatory amino acids are known to participate in this form of hippocampal structural plasticity [10], [13], [14]. Several experimental findings have been presented to account for this; for instance, stress-induced dendritic atrophy is prevented by NMDA receptor antagonists [13], [15], [16] and by the antiepileptic drug phenytoin [10], a molecule that also interferes with glutamatergic neurotransmission.

Not only are glutamatergic receptors and cytoskeletal proteins important for synapse structure, stability and function, but also cell adhesion molecules play a determinant part [17]–[21]. The neural cell adhesion molecule (NCAM) is one of the most abundant in hippocampal excitatory synapses [22], [23]. In addition to its role in synapse adhesion, robust evidence supports an NCAM role during synaptogenesis, recruiting and stabilizing the vesicular pool [24], [25]. On the other hand, PSA-NCAM (the polysialylated form of NCAM) has been implicated in neurite growth during synaptogenesis, synapse remodeling and plasticity [26], [27]. Interestingly, remodeling of hippocampal dendritic spines after polysialic acid (PSA) removal from NCAM has been recently described [28]. Nevertheless, it remains unclear whether NCAM and PSA-NCAM participate in dendritic retraction, and if so, to what extent they contribute to synaptic remodeling and/or disassembly.

The aim of the present work was thus to investigate the synaptic and dendritic events that take place during hippocampal dendritic retraction and atrophy. In particular, we studied whether defects in synaptic NCAM correlate with dendritic retraction and atrophy. Finally, we evaluated how synaptic remodeling is related to dendritic atrophy. As experimental model, we employed an in vitro approach which consisted of exposing cultured hippocampal neurons, after the synaptogenic peak, to a low glutamate concentration (5 µM), a strategy that resulted in a reduction of the dendritic tree area in the absence of neuronal death. It is worth noting that glutamate can facilitate or inhibit dendrite outgrowth, an effect that is time and concentration-dependent [29], [30]. In this setting, we studied the temporal course of events related to synaptic remodeling and dendritic retraction by evaluating the expression pattern of the pre-synaptic marker synaptophysin (SYN), the post-synaptic marker post-synaptic density 95 (PSD-95), and the cell adhesion molecules NCAM and PSA-NCAM. The temporal course of glutamate-induced dendritic retraction was studied in fixed cells as the area of MAP-2 positive structures and in living hippocampal neurons by means of cytoplasmic labeling.

## Materials and Methods

### Ethics statement

Experiments were carried out in accordance with the Guide for the Care and Use of Laboratory Animals provided by the NIH, USA. The experimental protocols were approved by the Ethics Committee for the Care and Use of Laboratory Animals of the School of Pharmacy and Biochemistry at the University of Buenos Aires (Approval Number: 220312-1). Special care was taken to minimize the number and suffering of the animals used.

### Animals and drugs

Adult Wistar rats (Facultad de Ciencias Exactas y Naturales, UBA) weighing 200–230 g at the beginning of the experiments were used. Animals were housed in groups of four in an air-conditioned room (temperature: 20±2°C) and maintained on a 12–12 h light/dark cycle. Food and water were available ad libitum.

All chemicals used were of analytical grade. Mouse monoclonal anti-NCAM (5B8) and anti-PSA-NCAM (5A5) were purchased from Hybridoma Bank (Iowa, USA). Mouse monoclonal anti-microtubule-associated protein 2 (MAP-2, Sigma Chemical Co.), anti-SYN (Chemicom), anti-PSD-95 (Affinity BioReangents Inc.), and secondary antibodies (FITC- and Red-labeled) (Jackson InmunoReasearch Laboratory Inc.) were used. Vectashield mounting medium was provided from Vector Laboratory Inc. All reagents used in culture including CellTracker Green CMFDA (5-chloromethyl fluorescein diacetate) were purchased from Invitrogen. Functional PSA mimetic peptide (fPSA, H- NTHTDPYIYPID- OH) was purchased from PB-L Products (University of Quilmes, Argentina). MK-801, or dizocilpine (5*S*,10*R*-(+)-5-Methyl-10,11-dihydro-5*H*-dibenzo[*a*,*d*]cyclohepten-5,10-imine maleate), 6-cyano-7-nitroquinoxaline-2,3-dione (CNQX) and Lipofectamine were purchased from Sigma-Aldrich (St Louis, MO, USA).

### Primary neuronal cultures and glutamate treatment

Pregnant Wistar rats on embryonic day 17–18 were placed in a CO_2_ chamber and then decapitated. The embryos were quickly removed by caesarean section and the hippocampi dissected and processed according to previous reports [31]. Hippocampal tissue was trypsinized, mechanically dissociated and plated on poly-D-lysine-coated glass coverslips at 8×10^3^ cells/cm^2^. Cells were maintained for 13–14 days in vitro (DIV) in Neurobasal medium supplemented with 2% (v/v) B27 and 0.5 mM glutamine. At 12–13 DIV, neurons were treated with 5 µM glutamate for 3 minutes at 37°C and media was immediately removed. Cells were washed with Hank's balanced salt solution, incubated in supplemented culture media for the indicated period of time, either 24 h or 1, 3, 6 or 12 h intervals for the time course experiments, and then fixed as described bellow for immunostaining. Control neurons were treated with vehicle, washed with Hank's balanced salt solution and identically processed and fixed (1, 3, 6, 12 or 24 h) as described above. In the experiments with glutamate receptor antagonists, neurons were pre-incubated before glutamate exposure with 10 µM MK-801 or 20 µM CNQX for 15 or 30 min, respectively. Experiments with fPSA were done according to previous reports. This PSA mimetic peptide has been shown to mimic PSA antigenic and biological properties [32]–[34]. Briefly, neurons were treated with 10 µM fPSA immediately after the glutamate treatment and incubated throughout the studied period (1, 3 or 6 h).

For live-imaging experiments, primary neuronal cultures were GFP-transfected (pmax-GFP 0.5 µg/µl, Amaxa) at 7 DIV using lipofectamine or stained with the CellTracker Green CMFDA (10 µM) 30 min before glutamate exposure. Cultures were also stimulated with 5 µM glutamate for 3 minutes, washed as described above and live-imaging recordings started 2 h later.

### Immunostaining

Neurons in culture (13–14 DIV) were fixed for 20 min at room temperature (RT) in 4% (w/v) paraformaldehyde/4% (w/v) sucrose in PBS solution, pH 7.2. After permeabilization with 0.2% (v/v) Triton X-100 for 10 min at RT and blockade with 5% (v/v) normal goat serum for 30 min at RT, neurons were incubated overnight at 4°C with primary monoclonal antibodies diluted in PBS (NCAM 1∶200; PSA-NCAM 1∶200; PSD-95 1∶200; SYN 1∶300 or MAP-2 1∶1000). The following day, cells were washed and incubated for 1 h at RT with the appropriate secondary antibodies. Hoechst 33342 (1∶2000) was used to stain the nuclei and determine the number of neurons with preserved nuclear morphology. Finally, cells were mounted using Vectashield mounting medium (Vector Laboratory Inc).

The cell surface staining was performed on water–ice slurry to reduce endocytosis, as previously described [31]. Neurons (13–14DIV) were washed with ice-cold Dulbecco's PBS (DPBS), incubated with PSA-NCAM antibody (1∶200) diluted in DPBS for 20 min, washed twice with DPBS, and fixed with 2% (w/v) paraformaldehyde in DPBS. Cells were then transferred to RT, washed twice with DPBS, incubated with red labeled secondary antibody for 30 min, washed three times with DPBS, permeabilized with 0.1% (v/v) Triton X-100 for 10 min, blocked, and immunostained for total NCAM as described above.

### Image acquisition

Immunofluorescence images from fixed cultures were captured employing 510–560 nm, 450–490 nm and 340–380 nm excitation filters in an Eclipse 50i Nikon epi-fluorescence microscope equipped with a Nikon DS-5M cooled camera. Images were taken to maximize dendritic and puncta staining. The analog images were digitized into an array of 2060×1920 pixels corresponding to an area of 311×233 µm of tissue (40× primary magnification). Morphology of cultured neurons was visualized by phase contrast microscopy. The analog images were digitized into an array of 1024×768 pixels corresponding to an area of 254×190 µm (20× primary magnification).

In time-lapse experiments and prior to placing the culture dish (with a glass-bottomed coverslip as base) on the microscope, the incubation chamber was equilibrated to 37°C and 5% CO_2_. Fluorescence images were captured every 5 min during 3 h using an Olympus Fluoview FV1000 laser scanning confocal microscope with a 60× PlanApo oil-immersion objective [1.4 numerical aperture (NA)] on an IX81 inverted microscope. Images of single optical sections through the neurite plane were acquired with 1.7 digital zoom and a 465 nm excitation Laser. For PSA-NCAM cell surface evaluation, single optical sections and compression series (projections) of *z*-axis optical sections were used.

Microphotograph compositions were prepared using the Adobe Photoshop CS3 software (Adobe Systems Inc.).

### Quantification

Digital image analysis was performed using the Image J analyzer sofware (NIH). Neuronal viability was quantified as the number of neurons per field of view (FOV = 280,000 µm^2^; 20× magnification) with normal Hoechst staining, and expressed as mean values (±SEM) of 10 microscope fields per experimental condition. The experiment was repeated at least three times. Dendritic tree area per neuron was calculated by subtracting the area corresponding to the immunolabeled soma from the total MAP-2 immunostaining. The number of puncta, total puncta area and individual puncta area for SYN, PSD-95 and NCAM were calculated as previously described [31], [35], [36]. For PSA-NCAM immunostaining [26], [37], total immunoreactive area per neuron was calculated.

The number of puncta, total puncta area and individual puncta area were calculated employing the count particle function of the ImageJ programme (NIH) according to previous reports [31]. Puncta were defined as any labelled punctum that represents clusters of SYN, PSD-95 and NCAM. For that purpose, a range of gray values (threshold values) was interactively selected to allow segmentation of the specific signal from the background, and thereafter did not differ within or across conditions. Thresholds were slightly adjusted between sections to maximize number of puncta and minimize the extent of fusion between puncta, but the values did not differ within or across conditions. Dendritic retraction was determined in terminal branches of four neurites per CMFDA-positive neuron, and was expressed as percentage of dendritic length [(length at 2 h-length at studied time)/length at 2 h×100]. Unless otherwise stated, all parameters are expressed as mean values (±SEM) of 20–30 neurons per experimental condition. Each immunostaining assay consisted of 1–2 coverslips per experimental condition. The experiments were repeated at least three times.

### Statistics analysis

Statistical analyses were performed using the InfoStat software. Statistical significance was set at P <0.05. Of the parameters studied 24 hours after glutamate exposure, dendritic tree area, SYN puncta number, total SYN puncta area, individual NCAM puncta area and total PSA-NCAM immunoreactive area were statistically analysed by non-parametric Mann-Whitney test, whereas PSD-95 and NCAM puncta number, total PDS-95 and NCAM puncta area and individual SYN and PDS-95 puncta area that were analyzed by Student *t* test. Percentage of dendritic length was analysed by Student *t* test. Results from experiments employing MK-801 or CNQX were respectively analysed by two-way and one-way analysis of variance (ANOVA) followed by Bonferroni test. All parameters obtained from the time course experiments were subjected to non-parametric Mann-Whitney test.

## Results

### Dendritic atrophy in the absence of neuronal death induced by brief exposure to a low glutamate concentration

**Figure 1:**
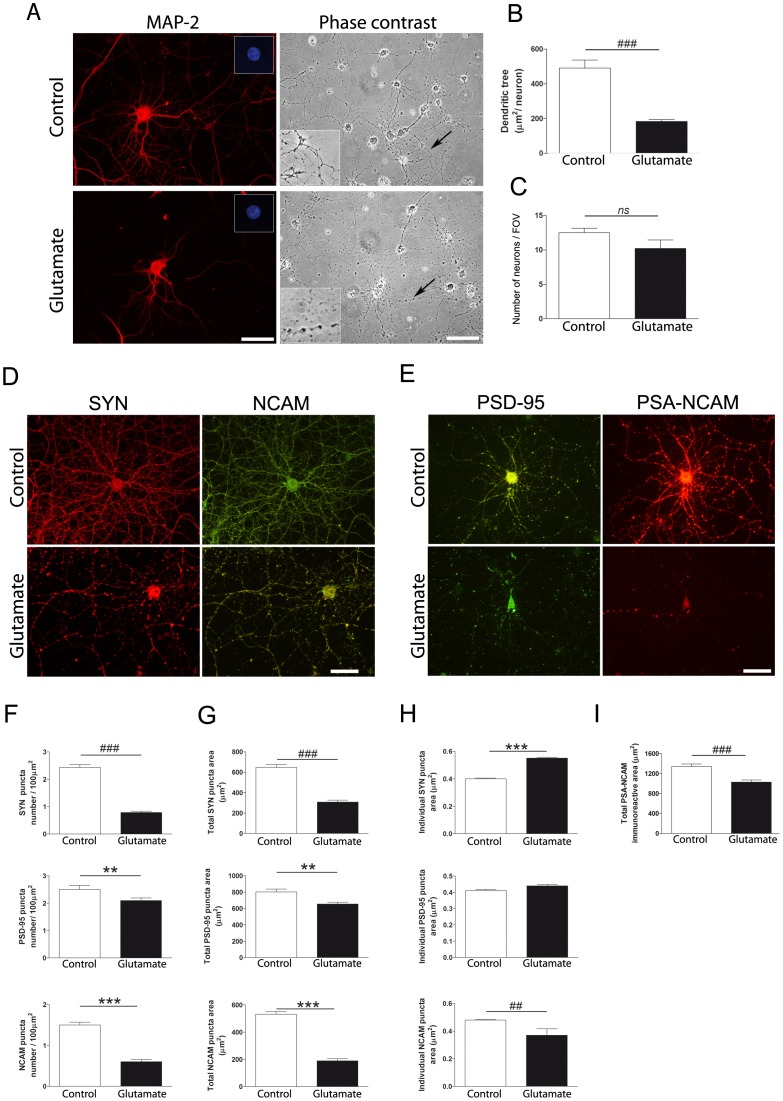
Glutamate exposure reduces MAP-2 immunostaining and induces changes in synaptic markers and cell adhesion molecule NCAM in the absence of neuronal death. Hippocampal neurons in culture (DIV 12–13) were briefly exposed to 5 µM glutamate and evaluated 24 h later. (A) Microphotographs of hippocampal neurons in culture immunostained for MAP-2 or visualized by phase contrast microscopy. Hoechst staining is shown in the upper right inset. (B) Quantification of MAP-2 immunostaining showed reduced dendritic tree area in glutamate-exposed neurons. (C) Glutamate exposure did not affect neuronal viability, quantified as the number of neurons per field of view (FOV) with normal Hoechst staining. Microphotographs of hippocampal neurons in culture doubled stained for (D) SYN and NCAM or (E) PSD-95 and PSA-NCAM. (F) Synaptic puncta number, (G) total synaptic puncta area and (H) individual puncta area were quantified for SYN, PSD-95 and NCAM. (I) Total immunoreactive area was measured for PSA-NCAM. Glutamate-exposed cultured hippocampal neurons showed decreased puncta number and area for SYN, PSD-95 and NCAM. Individual puncta area was larger for SYN, unchanged for PSD-95 and smaller for NCAM. PSA-NCAM immunoreactive area drastically decreased. Results are expressed as mean values (±SEM) of 20-30 neurons or 10 microscope fields (280,000 µm^2^/FOV) per experimental condition. *ns*, non-significant. ^##^ P<0.01 and ^###^ P<0.001 between bars, non-parametric Mann-Whitney test. ** P<0.01 and *** P<0.001 between bars, Student *t* test. MAP-2: microtubule-associated protein 2; NCAM: neural cell adhesion molecule; PSA-NCAM: polysialylated form of NCAM; PSD-95: post-synaptic density 95; SYN: synaptophysin. Scale bars: 50 µm and 100 µm for phase contrast.

Twenty-four hours after a brief exposure to a low glutamate concentration we found a reduction in the specific dendritic marker MAP-2 immunostaining (Fig. 1A), which rendered into a diminished neuronal dendritic tree area (Fig. 1B). However, glutamate treatment did not affect neuronal viability, as evidenced by the number of neurons with preserved nuclear morphology (Fig. 1A inset and C). Phase contrast microphotographs in Fig. 1A show a discontinuous pattern in the projections of glutamate-treated neurons in the absence of cytoplasmic vacuolar formation.

### Neuronal effects induced by glutamate exposure on synaptic markers and cell adhesion molecule NCAM

Cultured neurons exposed to glutamate in a condition that induced dendritic retraction but not cell death showed changes at the synaptic level 24 h after treatment. Immunostaining for the pre-synaptic marker SYN was remarkably affected in glutamated-treated neurons (Fig. 1D). Quantification of SYN puncta number and area revealed a dramatic decrease (Fig. 1F and G), with a concomitant increase in SYN individual puncta area (Fig. 1H). Similar results were found for the post-synaptic marker PSD-95 (Fig. 1E). However, reduction in PSD-95 puncta number and area (Fig. 1F and G) occurred in the absence of changes in PSD-95 individual puncta area (Fig. 1H).

Interestingly, glutamate-treated neurons exhibited changes in NCAM immunostaing 24 h after the insult (Fig. 1D). In this case, reduction in NCAM puncta number and area (Fig. 1F and G) was accompanied by a decrease in NCAM individual puncta area (Fig. 1H). Since PSA-NCAM does not form synaptic puncta due to its non-adhesive properties, only total immunoreactive area could be measured for this marker 24 h after neuronal exposure to glutamate, which revealed a pronounced decrease (Fig. 1E and I).

### NMDA receptor-dependent MAP-2 and PSA-NCAM reduction

**Figure 2:**
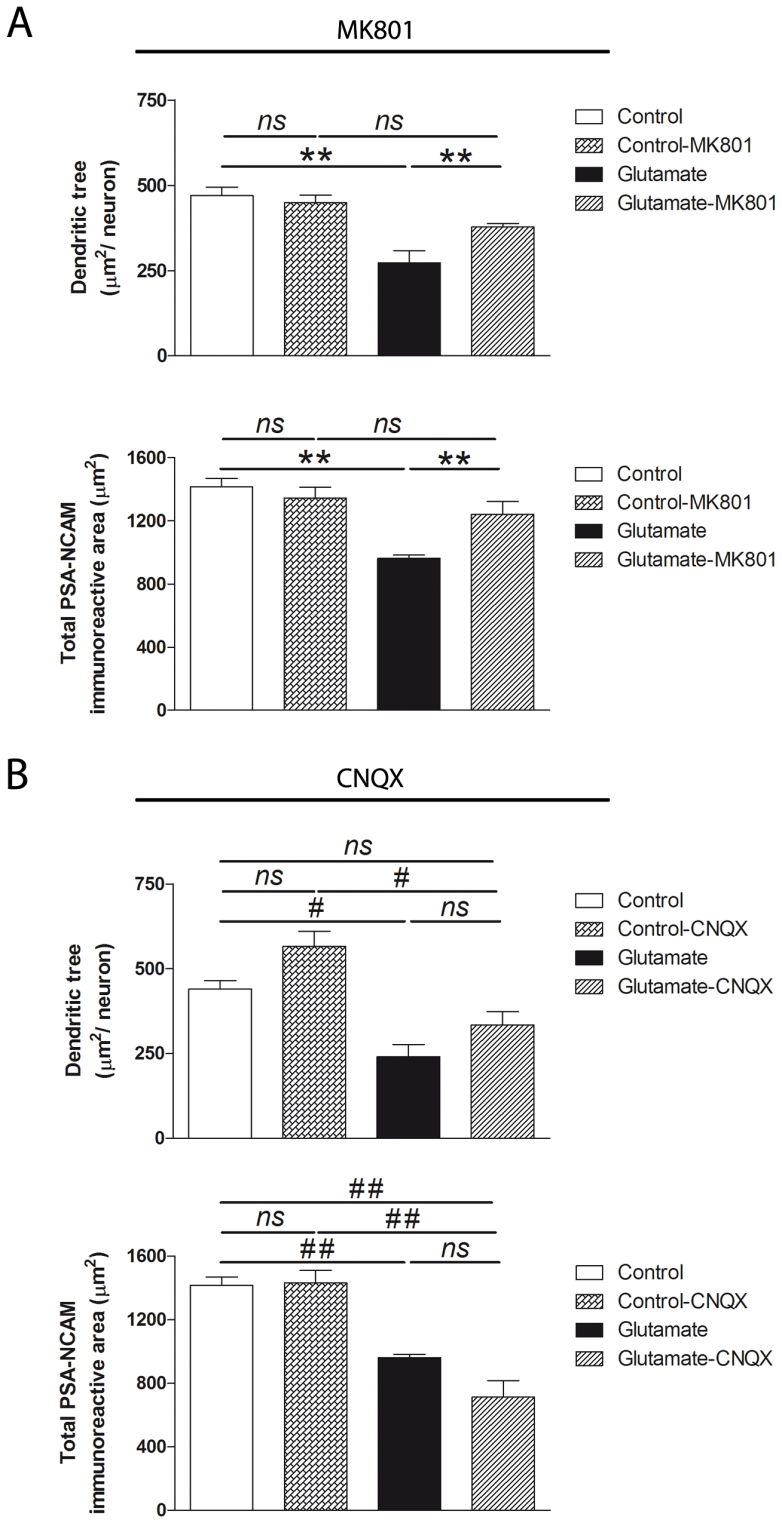
NMDA receptor blockade prevents MAP-2 and PSA-NCAM reduction induced by glutamate exposure. Hippocampal neurons in culture (DIV 12–13) were evaluated 24 h after a brief exposure to 5 µM glutamate in the absence and presence of different glutamate antagonists. (A) Quantification of MAP-2 and PSA-NCAM immunostainings showed reduced dendritic tree area and total PSA-NCAM immunoreactive area in glutamate-exposed neurons, two findings that were prevented by pre-treatment with 10 µM MK-801. (B) Reductions in dendritic tree and PSA-NCAM immunoreactive areas were not prevented by pre-treatment with 20 µM CNQX. MAP-2 and PSA-NCAM immunoreactive areas are expressed as mean values (±SEM) of 30 neurons per experimental condition. *ns*, non-significant; ** P<0.01 between bars, two-way analysis of variance (ANOVA) followed by Bonferroni test. *ns*, non-significant; ^#^P<0.05; ^##^P<0.01 between bars, one-way ANOVA followed by Bonferroni test. CNQX: 6-cyano-7-nitroquinoxaline-2,3-dione; MK-801: (5*S*,10*R*)-(+)-5-Methyl-10,11-dihydro-5*H*-dibenzo[*a*,*d*]cyclohepten-5,10-imine maleate or dizocilpine; PSA-NCAM: polysialylated form of NCAM.

In order to determine glutamate receptor specificity, neurons were incubated with the NMDA receptor antagonist MK-801 before glutamate exposure. In agreement with results described in Fig. 1, Fig. 2A shows that in the absence of the NMDA receptor antagonist, exposure to glutamate decreased neuronal dendritic tree area and reduced total PSA-NCAM immunostaining. Both glutamate-induced neuronal effects were prevented by MK-801 pre-treatment (Fig. 2A). On the contrary, reduction in the neuronal dendritic tree area and PSA-NCAM immunostaining was not prevented by pre-incubation with the AMPA receptor antagonist CNQX (Fig. 2B).

### Glutamate-dependent dendritic retraction and atrophy are relatively fast phenomena

To accurately define the temporal course of glutamate-induced dendritic retraction and atrophy, time-lapse experiments employing cytoplasmic markers were carried out.

**Figure 3:**
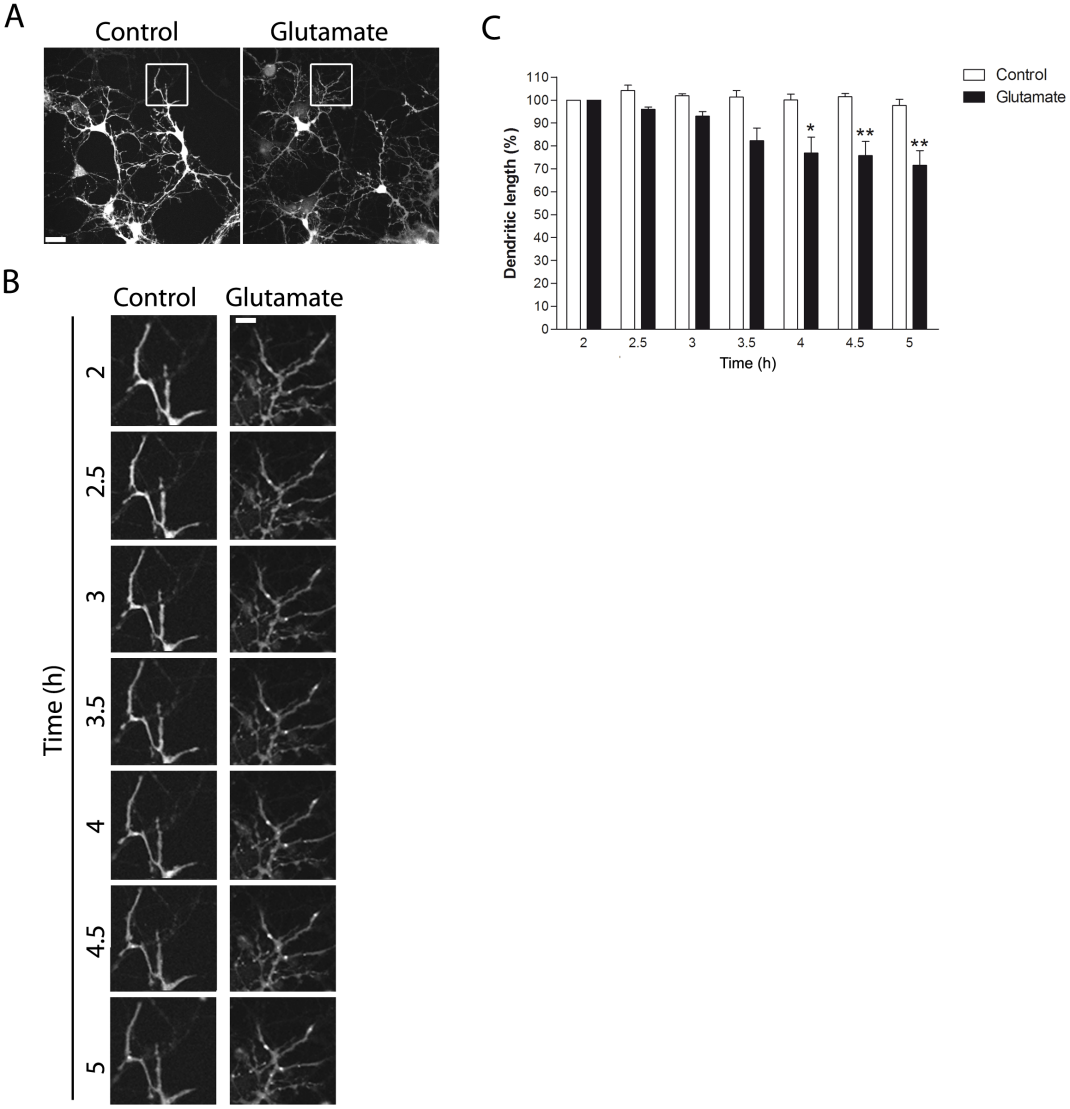
Glutamate-induced dendritic retraction visualized in CMFDA-stained neurons. (A) Hippocampal neurons in culture (DIV 12–13) were stained with the CMFDA dye and then briefly exposed to 5 µM glutamate, and live-imaged 2 h later every 5 min for a 3 h-period. (B) Microphotographs of hippocampal living neurons in culture showing the progressive shortening of CMFDA positive neurites. (C) Quantification of dendritic shortening over time. Dendritic retraction is shown as percentage (%) of the dendritic length measured 2 h after glutamate exposure. Dendritic retraction is clearly evident 4 h after glutamate exposure. Results are expressed as mean values (±SEM). * P<0.05 and ** P<0.01 versus 100%, Student *t* test. Scale bars  = 50 and 17 µm as magnification increases.

Glutamate-treated neurons previously filled up with the CMFDA dye showed a significant dendritic length decrease as early as 4 h after the insult (Fig. 3A and C). As shown in the microphotographs, magnification of the depicted area reveals shortening of the dendritic length after glutamate exposure (Fig. 3B and Videos S1 and S2).

**Figure 4:**
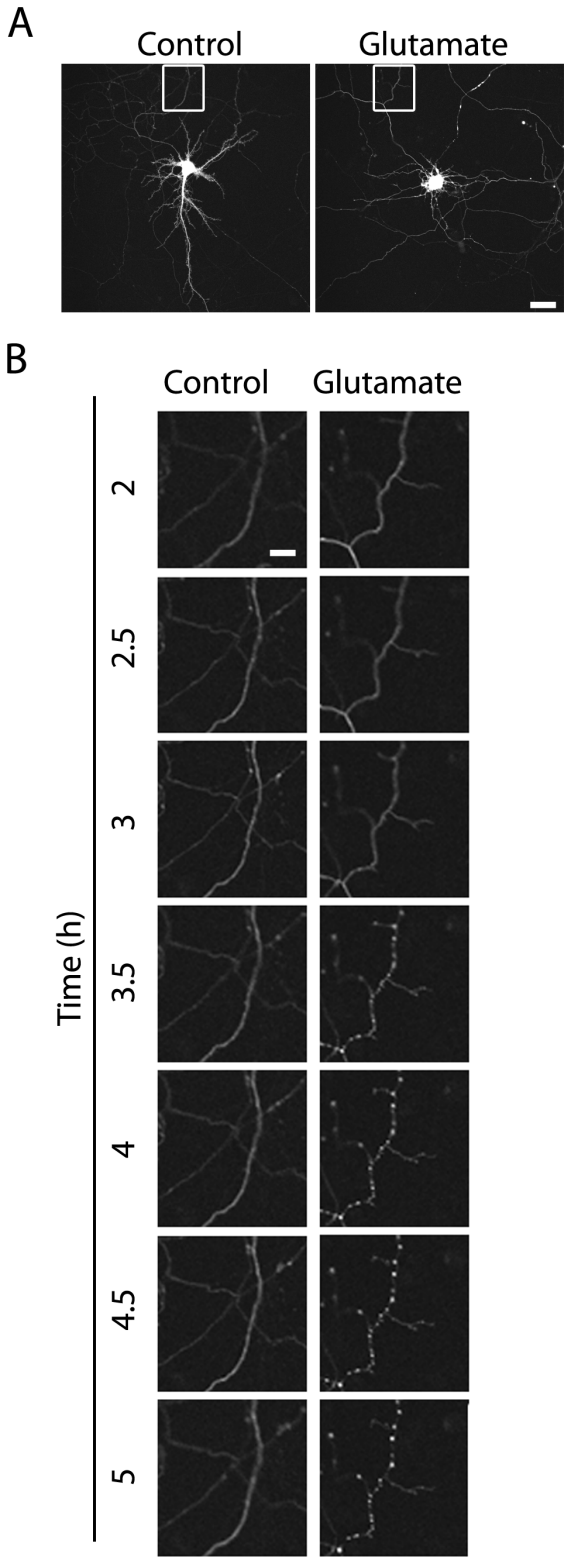
A continuous projection pattern becomes discontinuous after glutamate exposure. (A) GFP-transfected hippocampal neurons in culture (DIV 12–13) were briefly exposed to 5 µM glutamate and live-imaged 2 h later every 5 min for a 3 h-period.(B) Microphotographs of hippocampal living neurons in culture show the progression from a normal continuous pattern into a discontinuous one. Scale bars  = 50 and 13 µm as magnification increases.

Besides dendritic retraction and in agreement with Fig. 1A, GFP-transfected neurons treated with glutamate (Fig. 4A show that the classical continuous pattern observed in control projections became clearly discontinuous 3.5 h after glutamate treatment (Fig. 4B and Videos S3 and S4).

### PSA-NCAM decreases earlier than MAP-2 in response to glutamate exposure

**Figure 5:**
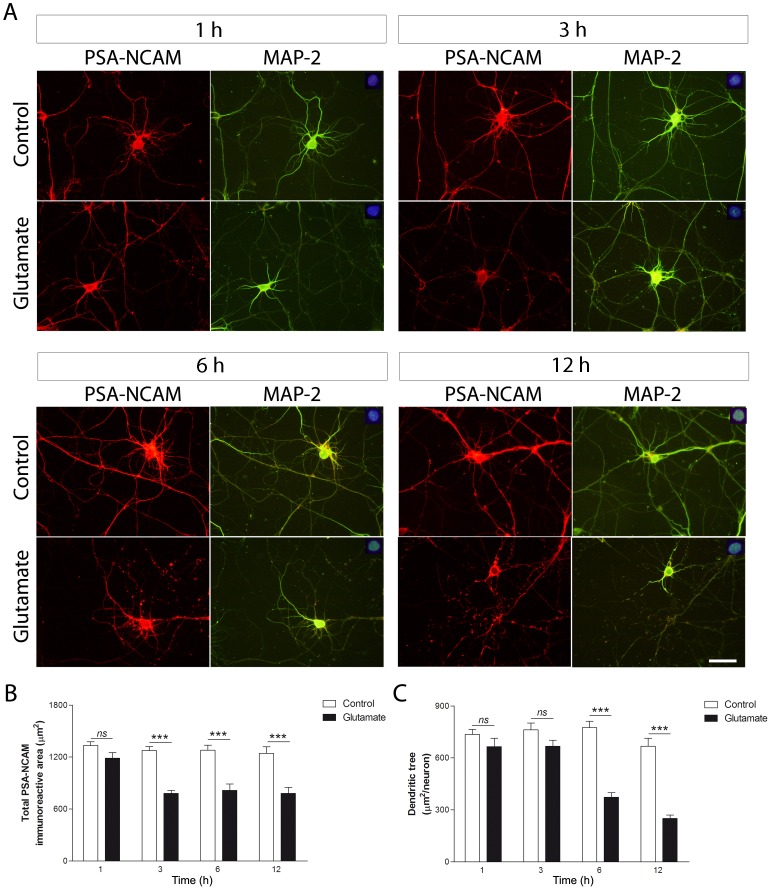
MAP-2 decrease follows the early PSA-NCAM reduction induced by glutamate exposure. Hippocampal neurons in culture (DIV 12–13) were briefly exposed to 5 µM glutamate and evaluated 1, 3, 6 and 12 h later. (A) Microphotographs of hippocampal neurons in culture doubled stained for MAP-2 and PSA-NCAM. (B) Quantification of total PSA-NCAM area showed decreased levels 3 h after glutamate exposure, which remained low for the rest of the studied period (3–12 h). (C) Quantification of MAP-2 immunostaining revealed that reduction in MAP-2 immunoreactive area follows PSA-NCAM diminution. MAP-2 and PSA-NCAM immunoreactive areas are expressed as mean values (±SEM) of 20–30 neurons per experimental condition. ns, non-significant; *** P<0.001 between bars, non-parametric Mann-Whitney test. MAP-2: microtubule-associated protein 2; PSA-NCAM: polysialylated form of NCAM. Scale bars: 50 µm.

Having found that dendritic retraction occurred approximately 4 h after neuronal exposure to glutamate, we then studied PSA-NCAM and MAP-2 immunorreactivity in adjacent time points, ranging from 1 to 12 h. Three hours after glutamate treatment, hippocampal neurons showed a decreased PSA-NCAM immunostaining that remained in lower values throughout the time points studied (3–12 h) (Fig. 5A). Instead, MAP-2 immunorreactivity reduction was not evident until 3 h later (6 h after glutamate exposure), and remained low through the rest of the studied period (12 h) (Fig. 5A). Quantification of total immunoreactive area for PSA-NCAM and MAP-2 confirmed that glutamate-induced PSA-NCAM reduction precedes the diminution in dendritic tree area (Fig. 5B and C).

**Figure 6:**
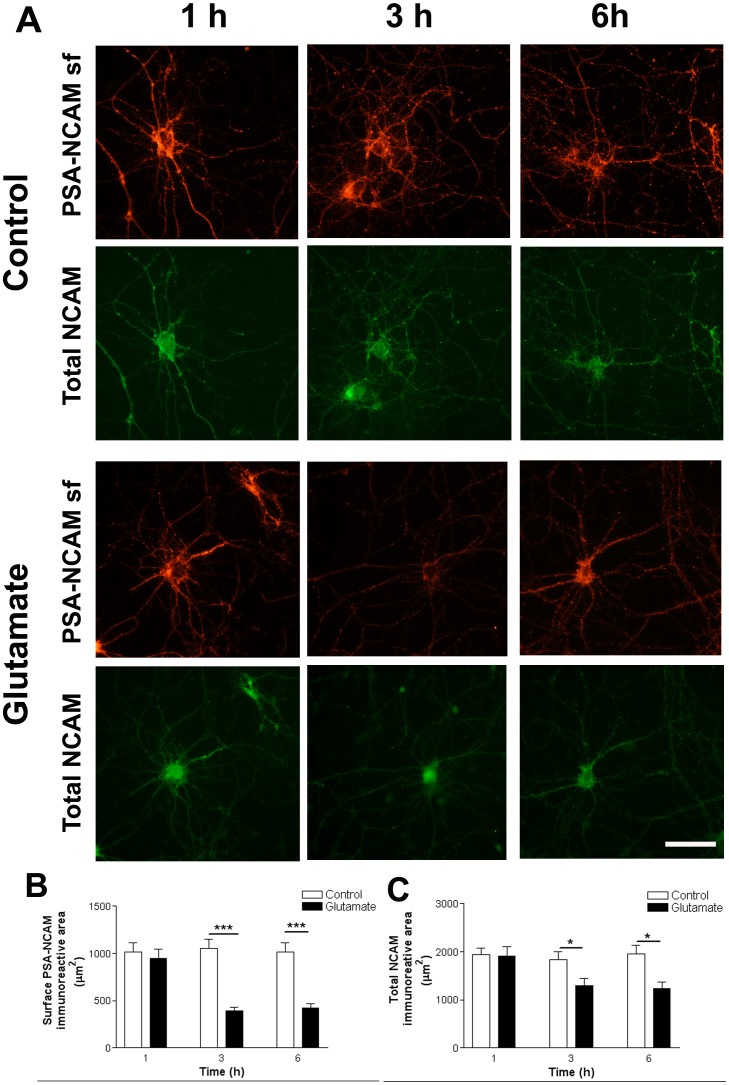
Early surface PSA-NCAM reduction induced by glutamate exposure. Hippocampal neurons in culture (DIV 12–13) were briefly exposed to 5 µM glutamate and evaluated 1, 3 and 6 h later. (A) Microphotographs of hippocampal neurons in culture doubled stained for surface PSA-NCAM and total NCAM. (B) Quantification of PSA-NCAM immunoreactive area showed decreased levels at neuronal surface 3 h after glutamate exposure, which remained low for the rest of the studied period (3–6 h). (C) Quantification of total (puncta plus diffuse staining) NCAM immunoreactive area revealed a concomitant NCAM reduction 3 h after glutamate treatment. Results are expressed as mean values (±SEM) of 20–30 neurons per experimental condition. * P<0.05, *** P<0.001 between bars, non-parametric Mann-Whitney test. NCAM: neural cell adhesion molecule; PSA-NCAM: polysialylated form of NCAM. Scale bar: 50 µm.

As described for total PSA-NCAM immunostaining (Fig. 5), PSA-NCAM reduction was also detected at neuronal surface 3 h after glutamate treatment. This effect lasted for at least 3 h and was accompanied with a reduction in NCAM immunostaining (Fig. 6A). Quantification of cell surface and total NCAM immunoreactive area confirmed glutamate-induced PSA-NCAM reduction at neuronal surface (Fig. 6B and C).

### NCAM shows a different time pattern in connection with glutamate-induced synapse remodeling and disassembly

**Figure 7:**
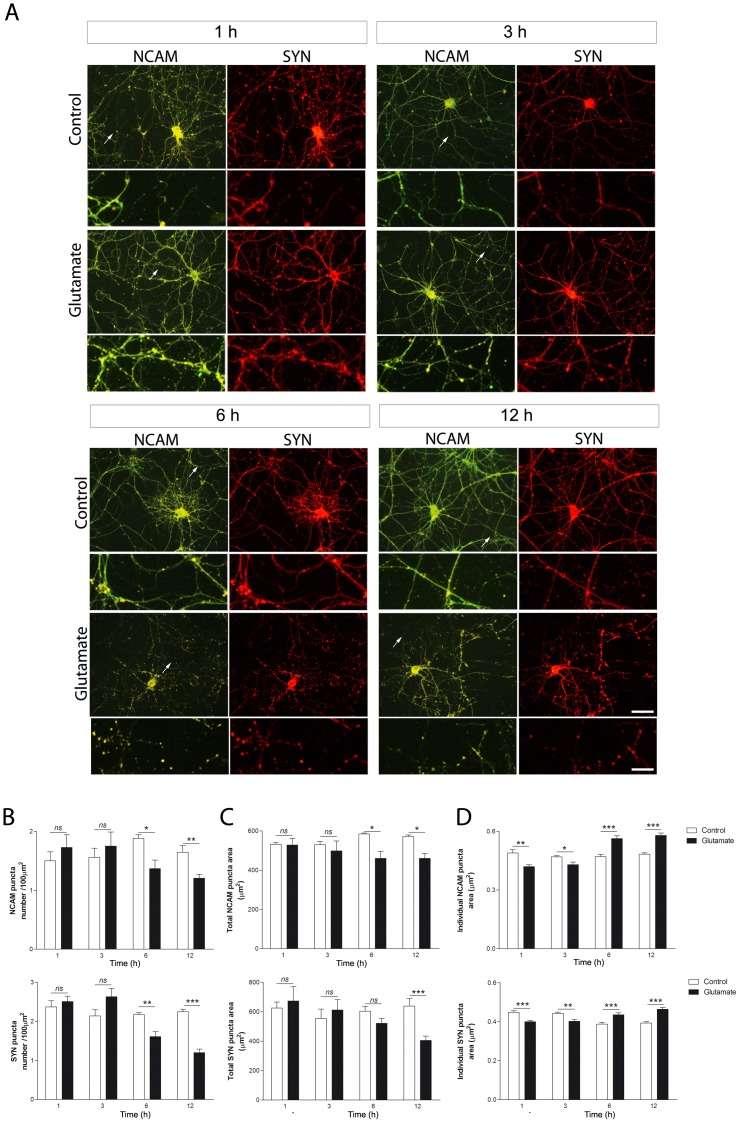
Time-course of changes induced on NCAM and SYN synaptic patterns and levels by glutamate exposure. Hippocampal neurons in culture (DIV 12–13) were briefly exposed to 5 µM glutamate and evaluated 1, 3, 6 and 12 h later. (A) Microphotographs of hippocampal neurons in culture doubled stained for NCAM and SYN. (B) Synaptic puncta number, (C) total synaptic puncta area and (D) individual puncta area were quantified for NCAM and SYN. Early decreases in individual NCAM and SYN puncta area precede the formation of larger NCAM and SYN clusters, evidenced as increased individual puncta sizes. This latter effect was accompanied by a reduction in the number of puncta and total puncta area for both NCAM and SYN. Results are expressed as mean values (±SEM) of 20–30 neurons per experimental condition. ns, non-significant; * P<0.05; ** P<0.01; *** P<0.001 between bars, by Mann-Whitney test. Scale bars: 50 µm and 10 µm as magnification increases. NCAM: neural cell adhesion molecule; SYN: synaptophysin.

Then, we studied glutamate-induced effects on NCAM and SYN expression patterns over time (Fig. 7). Diminution in individual NCAM puncta area became visible 1 h after glutamate treatment and remained decreased for at least 3 h (Fig. 7A and D). However, no changes were found in NCAM puncta number and total NCAM puncta area during this same period (1–3 h) (Fig. 7A, B and C). The same early pattern was observed for SYN; a diminution in individual SYN puncta area with no changes in SYN puncta number or total puncta area (Fig. 7A, B, C and D). Six hours after glutamate exposure, opposite effects were found on individual NCAM puncta area (Fig. 7A and D). On the other hand, total NCAM puncta number and area decreased (Fig. 7A, B and C). These effects lasted for at least 6 h. Synaptic SYN expression showed the same pattern as described for NCAM during this period (6–12 h), except for total SYN puncta area reduction, which did not reach statistical significance until time point 12 h after glutamate treatment (Fig. 7A, B, C and D).

### The fPSA prevents glutamate-induced dendritic atrophy and concomitant synapse remodeling

**Figure 8:**
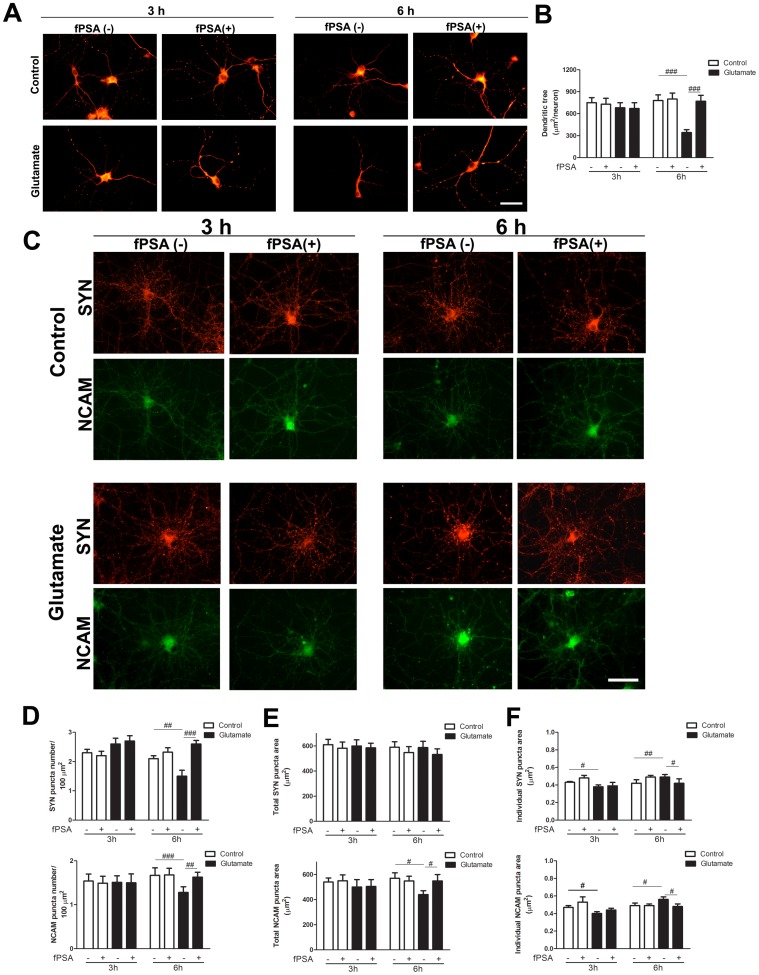
A functional PSA mimetic peptide (fPSA) prevents glutamate-induced MAP-2 decrease and concomitant changes in SYN and NCAM synaptic patterns and levels. Hippocampal neurons in culture (DIV 12–13) were briefly exposed to 5 µM glutamate, immediately treated with fPSA and evaluated 3 and 6 h later. (A) Microphotographs of hippocampal neurons in culture stained for MAP-2. (B) Quantification of MAP-2 immunoreactive area showed decreased levels 6 h after glutamate exposure; this decrease was prevented by fPSA. (C) Microphotographs of neurons double stained for SYN and NCAM. (D) Synaptic puncta number, (E) total synaptic puncta area and (F) individual puncta area were quantified for SYN and NCAM. The fPSA did not prevent early decrease in individual NCAM and SYN puncta area (3 h) but did prevent SYN and NCAM puncta changes seen 6 h after glutamate treatment. Results are expressed as mean values (±SEM) of 20–30 neurons per experimental condition. * P<0.05; ** P<0.01; *** P<0.001 between bars, by Mann-Whitney test. ^#^P<0.05; ^##^ P<0.01; ^###^ P<0.001 between bars, two-way ANOVA followed by Bonferroni test. Scale bars: 50 µm. fPSA: functional PSA mimetic peptide; MAP-2: microtubule-associated protein 2; NCAM: neural cell adhesion molecule; SYN: synaptophysin.

To test whether restoration of PSA actions would ameliorate the effects of glutamate, we added a functional PSA mimetic peptide (fPSA) immediately after glutamate treatment. fPSA prevented the decrease of MAP-2 immunoreactivity (6 h) (Fig. 8A). Quantification of MAP-2 immunoreactive area confirmed this result (Fig. 8B).

In our experimental conditions, fPSA did not affect any of the studied parameters in control conditions. While fPSA had no effect on early glutamate-induced SYN and NCAM puncta changes, particularly on the glutamate-induced individual puncta area reduction (3 h), addition of this PSA mimetic peptide prevented SYN and NCAM puncta changes that occurred 6 h after glutamate treatment (Fig. 8C–F).

## Discussion

It is well accepted that the hippocampus exhibits an impressive capacity for structural reorganization. Granule neurons of the DG and pyramidal neurons in areas CA3 and CA1 undergo dynamic modifications in the form of dendritic extension and retraction, as well as synapse formation and elimination. All of these types of structural plasticity are subject to modification by a variety of factors and conditions [1], [2]. Not surprisingly, dysfunctional structural plasticity in the form of reduced hippocampal volume, primarily due to dendritic atrophy, has been associated to several psychiatric and neurodegenerative disorders [6], [38], [39].

Herein, we demonstrate that a brief in vitro exposure of hippocampal neurons to a low glutamate concentration is capable of inducing dendritic atrophy in the absence of neuronal death, an effect that proved to be NMDA receptor-mediated. Indeed, our findings are in accordance with previous reports that show that in vivo administration of glutamatergic antagonists prevents the dendritic atrophy seen in chronic stress models [13], [15], [16]. Glutamate-induced dendritic retraction appears to be a gradual phenomenon that progresses to dendritic atrophy. Having determined that dendritic atrophy can be experimentally attained in a relatively short time-lapse (4 h in culture), we further investigated the earliest synaptic events, especially in connection with cell adhesion molecules.

Hippocampal cell adhesion molecules, in particular NCAM, which is one of the most abundant in excitatory synapses, are known to participate in synapse formation and stability [24], [25], [40], [41] but also in synaptic plasticity [27], [42]. PSA-NCAM promotes synapse formation and remodeling [26], [43]. Nevertheless, the role of the NCAM/PSA-NCAM balance in dendritic atrophy remains largely unknown. Interestingly, the hippocampus from NCAM (−/−) knockout mice is atrophic, notably in the CA3 subfield and the DG. The atrophy appeared to be due to reduced excrescence and loss of pyramidal cells in CA3, and reduced branching and volume of granule cell dendrites in the DG [44], [45]. Similarly, stress-induced dendritic atrophy has been associated to decreased NCAM hippocampal levels [46]–[50]. Although comparable results were obtained in our experimental model 24 h after glutamate treatment, upon studying the time course of early events we were able to determine that glutamate-induced dendritic atrophy is not preceded by reduction in synaptic NCAM levels. Our conclusion is based on the fact that the early reduction in NCAM individual puncta size is not accompanied by either decreased total NCAM puncta area or number. Instead, we found a tendency towards an increase in the number of NCAM puncta, which might indicate changes in the structure of the puncta adherentia [51].

As regards PSA-NCAM, increased levels of this molecule have been associated with synapse formation, neurite extension, repair and plasticity [26], [27], [48], [52]–[58]. Conversely, decreased PSA-NCAM levels have been related to stress-induced cognition deficits [59] and behavioral alterations in experimental depression [60]. Therefore, PSA-NCAM is nowadays considered a marker of structural synaptic remodeling [26], [43]. Surprisingly, we show that glutamate-induced dendritic atrophy is preceded by an important early decrease in PSA-NCAM levels at the neuronal surface. It is worth noting that PSA-NCAM levels can be down-regulated through endocytosis in different cell types including neurons [61], [62], a regulatory step that may affect PSA-NCAM levels at the plasma membrane.

Furthermore, it may be speculated that glutamate-induced dendritic atrophy is a consequence of early changes in the NCAM/PSA-NCAM balance. This imbalance would mainly affect the PSA-NCAM mediated events that control repair and promote neurite extension [51], [52], [55]–[57]. In accordance to this hypothesis, recent reports have described structural remodeling of hippocampal dendritic spines after PSA removal from NCAM [28] as well as changes in PSA-NCAM that parallel to dendritic retraction of amygdaloid interneurons after chronic stress [63].

Interestingly, we found a diminution in the number of synapses that occurs concomitantly with dendritic atrophy, as seen by the decreased puncta number and total puncta area for NCAM and SYN. These changes are accompanied by larger clusters of NCAM and SYN. The reported observations may well be the result of synaptic disassembly or endocytic transport processes. In fact, it is known that NCAM participates in the recruitment of the synaptic vesicle pool [24], [25] and that mono-ubiquitine is the signal that triggers its clathrin-dependent internalization [61], [64]. Therefore, our results depict a mechanism by which glutamate-dependent synaptic remodeling progresses to dendritic atrophy and synapse loss. Herein, we show that dendritic atrophy and synapse remodeling/disassembly are prevented by a fPSA, suggesting a PSA role in functional aspects of these processes. Moreover, our findings suggest that synaptic loss requires a reduction in PSA-NCAM as previously shown [26], but also implies changes in NCAM synaptic pattern and levels. The fact that PSA-NCAM has been shown to inhibit GluN2B-containing NMDA receptors at low glutamate concentrations [65], [66] may account for an enhanced NMDA-mediated glutamate response in a low PSA-NCAM condition such as the one found in our experimental model. It is noteworthy that PSA exerts a similar GluN2B-containing NMDA receptor inhibition. This effect has been proposed to be independent of the PSA carrier protein and to involve a direct or indirect action on this type of NMDA receptors [65]. Taking also into account that Torregrossa et al. (2004) [32] have suggested that fPSA adopts a conformation that mimics the PSA antigenic structure, it could be thought that a direct or indirect interaction of fPSA with GluN2B-containing NMDA receptors might take place in our conditions.

**Figure 9:**
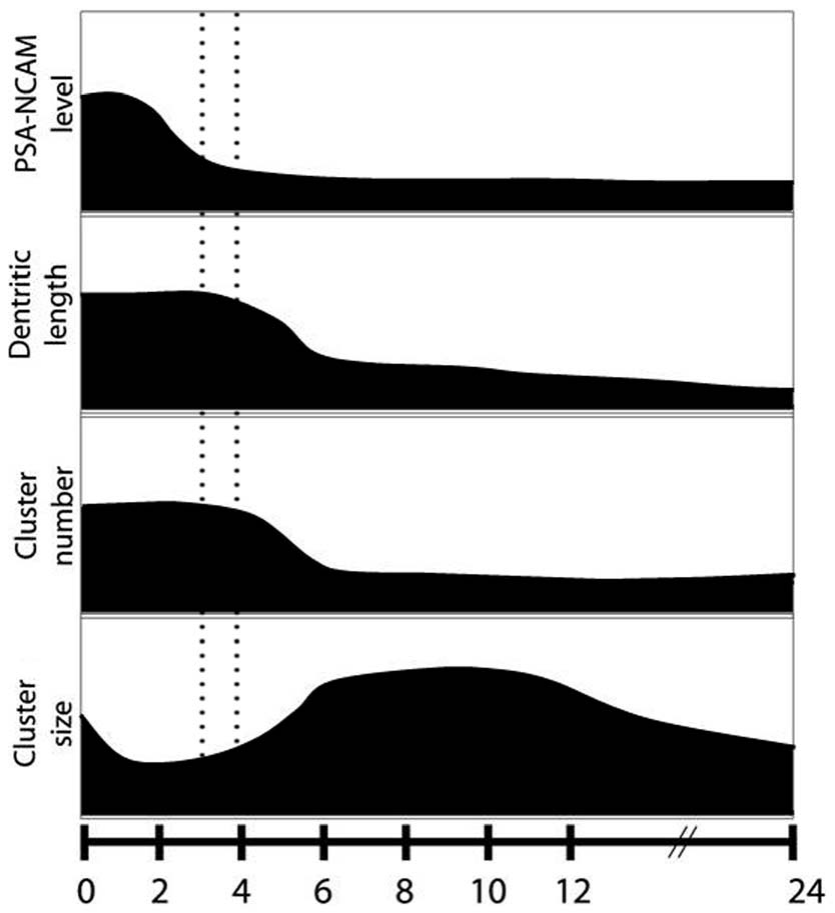
Schematic representation of glutamate induced changes on dendritic atrophy, NCAM and synaptic markers. Temporal course of changes induced by glutamate exposure reveals that NCAM-synaptic reorganization occurs soon after the insult and prior to the decrease in PSA-NCAM levels. Thereafter, dendritic atrophy, established 4 h after treatment, is followed by reductions in SYN and NCAM cluster number, which indicates synapse loss.

To sum up, our findings suggest that glutamate-dependent synaptic remodeling ultimately leads to dendritic atrophy and synapse loss. Moreover, they shed light on the time course of early events: while PSA-NCAM level diminution and NCAM-synaptic reorganization precede dendritic atrophy, synaptic-associated NCAM reduction is a delayed event related to synapse disassembly (Fig. 9). To our knowledge this is the first in vitro study to support the notion that distinctive stages in PSA-NCAM/NCAM balance accompany glutamate-induced dendritic atrophy and synapse loss. Not only altered PSA-NCAM levels have been reported in depression, schizophrenia and neurodegenerative disorders [60], [67]–[70], but also diminished NCAM levels were evidenced in some of these pathologies and in animal models of these diseases [45]–[49], [71], [72]. Hippocampal atrophy precedes synaptic and neuronal loss in a sequence of changes that could indicate different stages in the progression of the disease [37], [38], [73]. Therefore, it could be speculated that prevention or reversion of glutamate-induced atrophy could result in a successful early intervention. Further studies are required to shed light on the role of PSA-NCAM/NCAM balance in the prevention or reversion of brain pathologies that imply structural changes involving dendritic atrophy and synapse loss.

## Supporting Information

Video S1 **Glutamate-induced dendritic retraction visualized in CMFDA-stained neurons.** Hippocampal neurons in culture (DIV 12–13) were stained with the CMFDA dye and then briefly exposed to 5 µM glutamate, and live-imaged 2 h later every 5 min for a 3 h-period. Time-lapse series of control hippocampal living neurons in culture showing the normal progress of dendritic outgrowth and retraction in CMFDA positive neurites.
(WMV)

Video S2 **Glutamate-induced dendritic retraction visualized in CMFDA-stained neurons.** Hippocampal neurons in culture (DIV 12–13) were stained with the CMFDA dye and then briefly exposed to 5 µM glutamate, and live-imaged 2 h later every 5 min for a 3 h-period. Time-lapse series of glutamate-treated hippocampal living neurons in culture showing the progressive shortening of CMFDA positive neurites.
(WMV)

Video S3 **A continuous projection pattern becomes discontinuous after glutamate exposure.** GFP-transfected hippocampal neurons in culture (DIV 12–13) were briefly exposed to 5 µM glutamate and live-imaged 2 h later every 5 min for a 3 h-period. Time-lapse series of control hippocampal living neurons in culture showing the normal continuous projection pattern.
(WMV)

Video S4 **A continuous projection pattern becomes discontinuous after glutamate exposure.** GFP-transfected hippocampal neurons in culture (DIV 12–13) were briefly exposed to 5 µM glutamate and live-imaged 2 h later every 5 min for a 3 h-period. Time-lapse series of glutamate-treated hippocampal living neurons in culture showing the progression from a normal continuous pattern into a discontinuous one.
(WMV)
